# Preoperative FDG-PET/CT Is an Important Tool in the Management of Patients with Thick (T4) Melanoma

**DOI:** 10.1155/2012/614349

**Published:** 2012-05-13

**Authors:** Rodrigo Arrangoiz, Pavlos Papavasiliou, Carrie A. Stransky, Jian Q. Yu, Li Tianyu, Elin R. Sigurdson, Adam C. Berger, Jeffrey M. Farma

**Affiliations:** ^1^Department of Surgical Oncology, Fox Chase Cancer Center, 333 Cottman Avenue, Philadelphia, PA 19111, USA; ^2^Department of Surgery, Thomas Jefferson University, Philadelphia, PA 19107, USA; ^3^Department of Nuclear Medicine, Fox Chase Cancer Center, 333 Cottman Avenue, Philadelphia, PA 19111, USA; ^4^Department of Biostatistics, Fox Chase Cancer Center, 333 Cottman Avenue, Philadelphia, PA 19111, USA

## Abstract

The yield of preoperative PET/CT (PET/CT) for regional and distant metastases for thin/intermediate thickness melanoma is low. Objective of this study is to determine if PET/CT performed for T4 melanomas helps guide management and alter treatment plans. *Methods.* Retrospective cohort of 216 patients with T4 melanomas treated at two tertiary institutions. Fifty-six patients met our inclusion criteria (T4 lesion, PET/CT and no clinical evidence of metastatic disease). *Results.* Fifty-six patients (M: 32, F: 24) with median tumor thickness of 6 mm were identified. PET/CT recognized twelve with regional and four patients with metastatic disease. Melanoma-related treatment plan was altered in 11% of the cases based on PET/CT findings. PET/CT was negative 60% of the time, in 35% of the cases; it identified incidental findings that required further evaluation. *Conclusion.* Patients with T4 lesions, PET/CT changed the treatment plan 18% of the time. Regional findings changed the surgical treatment plan in 11% and the adjuvant plan in 7% of our cases due to the finding of metastatic disease. Additionally 20 patients had incidental findings that required further workup. In this subset of patients, we feel there is a benefit to PET/CT, and further studies should be performed to validate our findings.

## 1. Introduction

Cutaneous malignancies constitute one of the most commonly diagnosed cancers in the United States (USA), comprising more than half of all cancers diagnosed each year [1]. Cutaneous melanoma represents approximately four percent of all skin cancers diagnosed each year, but accounts for approximately 75% of skin-cancer-related deaths. In the USA in the year 2010, 68,130 new cases of melanoma were diagnosed, with 8,700 patients dying of the disease [2]. The incidence is increasing dramatically. The age-adjusted incidence of invasive melanoma in the USA increased from approximately four per 100,000 to 18 per 100,000 in white males between 1973 and 1998 [3]. The incidence of cutaneous melanoma continues to increase dramatically, increasing in men more rapidly than any other malignancy, and in women more rapidly than any other malignancy except for lung cancer [4]. The lifetime risk of developing melanoma for someone born in the USA in the year 2000 is 1 in 41 for men and 1 in 61 for women [3].

18-Fluorodeoxyglucose (^18^F-FDG) positron emission tomography/computed tomography (PET/CT) is a useful functional imaging modality for the staging of melanoma and has a potential role in assessing response to therapy. Wahl et al. [5] and Kern [6] demonstrated that radiolabeled glucose analogs were preferentially taken up in murine melanomas and human melanoma xenografts, setting forth the rationale for the potential use of FDG in patients with melanoma.

Numerous studies have shown that PET/CT is not useful in the initial staging of thin and intermediate thickness primary cutaneous melanoma when there is no clinical evidence of local or distant metastatic spread [7–10]. The reason for this is the small size/volume of most nodal metastases combined with the low prevalence of nodal disease in patients with early primary melanomas [11]. PET/CT generally can only detect lesions approximately 8 mm in diameter. Friedman and Wahl recommend using PET/CT for evaluating patients with cutaneous melanoma who fit into one of the four following categories: (1) individuals with a high risk for distant metastases based on extent of locoregional disease, (2) patients with findings that are suspicious for distant metastases, (3) individuals with known distant tumor deposits who still stand to benefit from customized therapies if new lesions are discovered or treated lesions regress, and (4) patients at high risk for systemic relapse who are considering aggressive medical therapy [12]. The objective of our study was to determine if preoperative PET/CT performed for T4 melanomas helped guide management and alter the treatment plan. 

## 2. Methods

A retrospective cohort study was performed at two tertiary referral centers, Fox Chase Cancer Center and Thomas Jefferson University Hospital. Institutional review board approval was obtained from both institutions. Inclusion criteria were as follows: patients with the histopathologic diagnosis of a primary cutaneous melanoma with a T4 (tumor thickness greater than 4 mm) lesion based on the AJCC staging system [13], patients who had a preoperative PET/CT performed as part of their staging workup, and no clinical evidence of locoregional or distant metastatic disease. We identified 216 patients with T4 melanomas treated between January 2003 and January 2009. Fifty six patients met our inclusion criteria of having a T4 lesion with a preoperative PET/CT and no clinical evidence of regional or distant metastatic disease. Medical records were reviewed, and patient demographics, characteristics of their primary tumor, and findings from PET/CT studies were analyzed. Any deviation from the preoperative plan (change in the surgical procedure, addition studies for the workup of incidental findings, or additional consultations with the medical or radiation oncologist) that occurred due to the results of the staging PET/CT was considered as a change in the treatment plan. A true positive PET/CT for regional disease and metastatic disease was defined by the presence of malignant disease in the final pathology specimen and during the workup of metastatic disease (ultrasound-guided or CT-guided biopsies of the metastatic foci).

The PET/CT scans were obtained according to the following standard protocol in both institutions: all patients were asked to fast for at least 4 hours before the study. After measurement of blood glucose level and confirming it is below 200, the patient was given an I.V. injection of approximately 15 mCi of ^18^F-FDG as standard dose. After a delay of 1 to 2 hours, the patient voided, and the PET/CT scan was performed on a GE Discovery LS PET/CT Scanner or a Siemens Biograph 16 PET/CT Scanner from the vertex of the head down to feet for all patients to cover the entire skin surface. The CT images were acquired in helical mode during normal breathing and were used for registration with the PET images and for applying attenuation correction.

For Discovery LS (from 2003 to 10/2010), the CT scan acquisition parameters were 140 kVp, 90 mA, 0.8 s per rotation, slice thickness of 5 mm, 0.75 pitch, and interval of 4.25 mm. The field of view for PET and CT images was 50 cm diameter. The PET scans were acquired for 6 or 7 minutes per bed position in 2D mode with a single-slice overlap and were reconstructed using ordered subsets expectation maximization (OSEM) algorithm with 28 subsets and 2 iterations using manufacturer-supplied software. The PET system has a 2-dimensional transaxial resolution of 4.7 mm full width half maximum (FWHM) at 1 cm radius and 5.2 mm at 10 cm radius.

For Siemens Biograph (from 10/2010 to present), the CT scan was acquired using CareDose4D with the following acquisition parameters: 130 kVp, reference mAs of 100, 0.6 s per rotation, 5 mm slice thickness, pitch of 1.0, and 70 cm diameter field of view. The PET scan was acquired for 2 to 3 minutes per bed position in 3D mode with 16-slice overlap and was reconstructed using TrueX algorithm with 21 subsets and 2 iterations, with 63 cm diameter field of view using manufacturer-supplied software. The PET system has a 2-dimensional transaxial resolution of 4.4 mm FWHM at 1 cm radius and 4.6 mm at 10 cm radius.

The PET reconstruction included corrections for random and scatter. Attenuation correction was applied based on the low-dose CT to reduce radiation exposure to the patients. All images were corrected for body weight, dose administered, and radioactive decay and displayed on an eNTEGRA or Xeleris workstation for GE scanner or multimodality workplace for Siemens scanner with an initial standardized uptake value (SUV) gray scale of 0 (white) to 5 (black).

The PET scans were reviewed to determine the length of the abnormality with an SUV of 2.5 as cutoff value to delineate the tumor extent. The maximum SUV within the tumor volume was also determined by manufacturer build-in computer algorithm.

The SUV values are comparable between the two scanners with cross-calibration performed by manufacturer-trained field engineers and our in-house medical physicist. There are many studies displayed and reviewed in both systems during the transition period from GE to Siemens scanner.

## 3. Results

Of the 56 patients, 32 (57%) were male and 24 (43%) were female. The mean age of the patients in our study was 67 years (range, 26 to 89 years), and the median tumor thickness was 6 mm (range, 4.1 mm to 40 mm). Tumor ulceration was identified in 61% of the cases (*n* = 34) and satellitosis in 25% (*n* = 25) of the cases. Fifty percent of the melanomas were located in the extremities (*n* = 28), 29% in the trunk (*n* = 16), and 21% in the head and neck region (*n* = 12) (Table 1).

**Figure 1:**
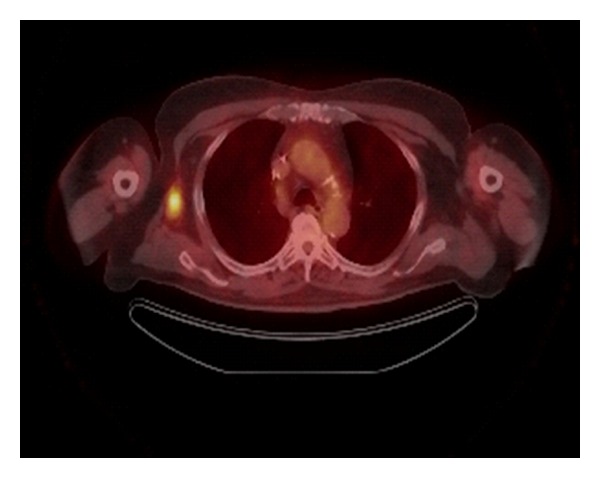
Lymph node uptake.

**Figure 2:**
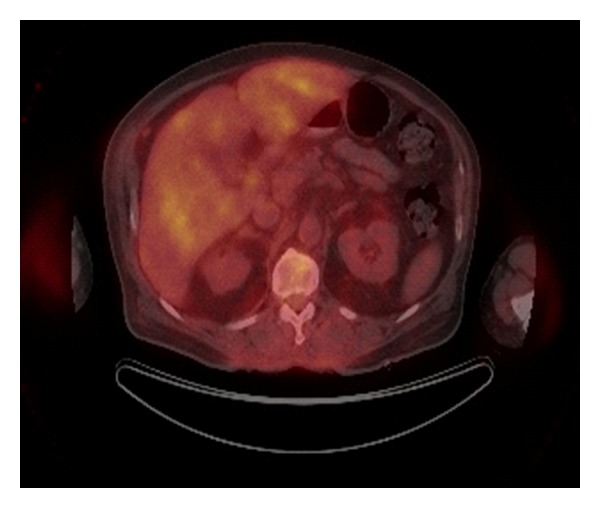
Liver and vertebral uptake.

**Figure 3:**
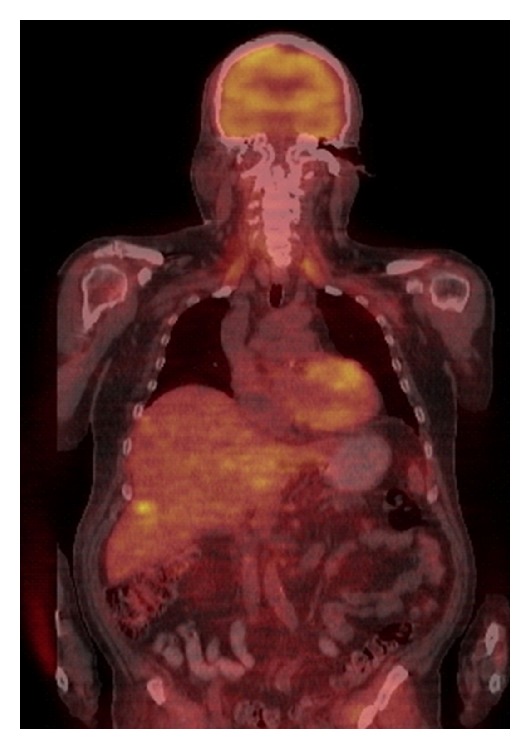
Liver uptake.

Preoperative PET/CT identified twelve (21%) patients with regional disease and four (7%) patients with metastatic disease (Figures 1, 2, and 3). Preoperative PET/CT changed the treatment plan 18% of the time. Regional findings changed the surgical treatment plan in 11% of our cases, and systemic chemotherapy (temozolomide) was added in 7% of our cases due to the finding of metastatic disease (Table 2). Six patients (11%) proceeded directly to therapeutic lymph node dissection due to the preoperative PET/CT findings, thereby eliminating the need for sentinel lymph node biopsy (SLNB). In all six cases, the reason for performing the therapeutic lymph node dissection rather than the SLNB was the depth of the primary melanoma, the presence of clinically detected lymphadenopathy, and the presence of PET/CT avid lymph nodes (patient number six did not undergo SLNB do to clinically evident disease in the inguinal region). Based on the findings of metastatic disease on the preoperative PET/CT, four patients were presented in a multidisciplinary tumor board for discussion of systemic chemotherapy and the possibility of having been enrolled into a clinical trial. Patient number three in Table 2 with metastatic disease in the liver underwent operative intervention with right axillary dissection for debulking and palliation only not for curative intent.

PET/CT was negative 60% (*n* = 33) of the time, and in 35% (*n* = 20) of the cases, it identified incidental findings that required further evaluation (mediastinal lymphadenopathy, lung, colon, and thyroid lesions). Three patients with positive findings on preoperative PET/CT were found to have incidental findings not related to their melanoma and form part of the 20 patients who were reported to have incidental findings. The sensitivity and specificity of PET/CT for identifying regional metastatic disease were 40% and 90%, respectively. The sensitivity and specificity for identifying distant metastatic disease were 100% and 94%, respectively (Table 3). The combined sensitivity and specificity for identifying regional and metastatic disease were 45% and 88%, respectively (Table 3). The positive predictive value (PPV) and negative predictive value (NPV) of PET/CT for identifying regional metastatic disease were 86% and 50%, respectively. Tables 4, 5, and 6 show the 2×2 tables that were used to calculate the sensitivities and specificities previously sited. The positive predictive value (PPV) and negative predictive value (NPV) of PET/CT for identifying distant metastatic disease were 57% and 100%, respectively. The combined positive predictive value (PPV) and negative predictive value (NPV) of PET/CT for identifying regional and metastatic disease were 82% and 55%. Of the 23 patients (48%) that had a positive SLNB, six (26%) had avid lymph nodes on PET/CT.

## 4. Discussion

The staging of patients with melanoma is a crucial step in the sequence of events that leads to instituting appropriate management. Multiple studies have shown that PET/CT has no role in the initial staging of thin and intermediate thickness melanomas in the absence of signs or symptoms suggestive of distant disease [11, 13, 14]. One of the first articles to suggest that PET/CT was not as sensitive for staging of regional nodes in patients with cutaneous melanomas came from a prospective trial containing 70 patients with primary melanomas (>1.0 mm in thickness) and four patients with recurrent melanoma who underwent PET/CT and sentinel lymph node (SLN) biopsy. The biopsy results were used as a gold standard for regional lymph node metastases. PET/CT scans found two true positives, 71 true negatives, 0 false positives, and 16 false negatives for a sensitivity of 11% and specificity of 100%. In this study, Wagner et al. concluded that PET/CT is an insensitive indicator of occult regional lymph node metastases in patients with melanoma because of the minute tumor volumes in this population [11]. In another study, Belhocine et al. [15] showed that of six histologically positive SLNs in patients with clinically localized disease, PET/CT identified only one metastatic focus in an SLN that was greater than one centimeter [16]. Acland et al. found that in 50 patients who underwent SLN biopsy for melanomas that were pathologically greater than 1 mm in thickness or invading lymphatic's, PET/CT failed to identify all 14 positive SLNs [17]. Another study of 609 patients staged using PET/CT (pooled from several studies), 38 patients (6%) had abnormal uptake outside the primary site or regional nodes, but only 1 had a true melanoma metastasis [16].

In our study of T4 melanoma, 12 (21%) patients had regional metastatic disease, and four (7%) patients had distant metastatic disease that was identified with preoperative PET/CT. The diagnostic yield of preoperative PET/CT for regional [8, 9, 11, 18–21] and distant metastases [18–22] for thin and intermediate thickness melanoma is low. A previous study from our institution retrospectively reviewed 83 patients with intermediate thickness melanoma who underwent SLN biopsy, of which 45% had preoperative PET/CT. Only two patients from this study with positive SLNB were found to have avid lymph nodes on preoperative PET/CT (Table 7). This study did not support the routine use of PET/CT for patients undergoing SLNB for melanoma [23]. Our study showed that the sensitivity and specificity of PET/CT for identifying regional metastatic disease were 40% and 90%, respectively. In the literature, the sensitivity and specificity of PET/CT for identifying regional metastatic disease have varied from 8% to 100% and 84% to 100%, respectively [8, 9, 11, 17, 24–26]. Studies performed in the 1990s have much better sensitivities compared to more recent ones, and this might be due to the small number of cases reported in the earlier studies, and possible selection/inclusion bias. The sensitivity and specificity of PET/CT for identifying distant metastatic disease in the literature have ranged from 78% to 100% and 22% to 87%, respectively [18–22]. In our study, the sensitivity and specificity for identifying distant metastatic disease were 100% and 94%, respectively. PET/CT findings did contribute important information that led to the modification of the original treatment plan and discovered an incidental finding in 20 cases that required further evaluation.

Some limitations to this study must be considered. This study represents a retrospective analysis from a cohort of patients from two tertiary institutes, and the possibility of selection bias always exists. Also due to the small sample size, we cannot make definitive conclusions on the potential benefits that PET/CT has on the initial evaluation of cutaneous melanoma.

Our study suggests that patients with thick melanomas can benefit from a preoperative PET/CT. The treatment plan of our patients was modified 18% of the time. In this subset of patients, preoperative PET/CT should be considered as part of the staging workup, and further studies should be performed to validate our findings.

## Figures and Tables

**Table 1:** Demographics.

Patient characteristics	
Patients (*n*)	56
Male: female (*n*)	32 : 24
Age (mean, range)	67 yrs, (26–89 yrs)

Tumor characteristics	
Median tumor thickness	6 mm, (4.1–40 mm)
Mean tumor thickness	9 mm
Tumor ulceration (*n*)	34
Satellitosis (*n*)	25

Location of the melanoma (*n*, % of cases)	
Trunk	16 (29%)
Extremities	28 (50%)
Head and neck	12 (21%)

**Table 2:** Altered surgical plan based on PET.

	Diagnosis		Operative plan	
Patient	Pre-PET scan	Post-PET scan	Pre-PET scan	Post-PET scan
1	Back melanoma	Axillary uptake	WLE + SLNB	WLE + L SLB +R LND
2	Back melanoma	Axillary uptake	WLE + SLNB	WLE + bilateral LND
3	Back melanoma	L Hilar, R axillary, and liver uptake	WLE + SLNB	WLE + R LND
4	Forehead melanoma	Submandibular, periauricular uptake	WLE + SLNB	WLE + parotidectomy + R MRND
5	Leg melanoma	Inguinal node uptake	WLE + SLNB	WLE + LND
6	Foot melanoma	uptake at calf	WLE + SLNB	WLE + R LND + excision of In transit lesion

WLE = wide local excision; SLNB = sentinel lymph node biopsy; LND = lymph node dissection; MRND = modified radical neck dissection; R = right; L = left.

**Table 3:** Sensitivity and specificity.

	Sensitivity	Specificity
Combined	0.5	0.88
Regional	0.4	0.9
Mets	1.0	0.94

**Table 4:** 2 × 2 Table of PET/CT with regional metastatic disease.

+ PET/CT with regional Disease	Confirmed regional disease		
Frequency percent			
	(a) Disease present	(b) No Disease	Total
(a) Positive test	14	2	16
	23.08	3.85	26.92
(b) Negative test	19	21	40
	34.62	38.46	73.08
Total	33	22	56
	57.69	42.31	100.00

**Table 5:** 2 × 2 Table of PET/CT with distant metastatic disease.

+ PET/CT with metastatic disease	Confirmed metastatic disease		
Frequency percent			
	(a) Disease present	(b) No Disease	Total
(a) Positive test	5	3	8
	7.27	5.45	12.73
(b) Negative test	0	48	48
	0.00	87.27	87.27
Total	5	51	56
	7.27	92.73	100.00

**Table 6:** 2 × 2 Table of PET/CT with combined regional and distant metastatic disease.

+ PET/CT regional and metastatic disease	Confirmed regional and metastatic disease		
Frequency percent			
	(a) Disease present	(b) No disease	Total
(a) Positive test	15	3	18
	25.45	5.45	30.91
(b) Negative test	17	21	38
	30.91	38.18	69.09
Total	32	24	56
	56.36	43.64	100.00

**Table 7:** Sentinel Lymph Node Biopsy (SLNB).

	Yes	No
SLNB	48 (86%)	6 (11%)
Positive SLNB	23 (48%)	25 (52%)
Positive SLN and a positive node on PET/CT	6 (26%)	17 (74%)
